# Health Care Services Utilization of Persons with Direct, Indirect and without Migration Background in Germany: A Longitudinal Study Based on the German Socio-Economic Panel (SOEP)

**DOI:** 10.3390/ijerph182111640

**Published:** 2021-11-05

**Authors:** Thomas Grochtdreis, Hans-Helmut König, Judith Dams

**Affiliations:** Hamburg Center for Health Economics, Department of Health Economics and Health Services Research, University Medical Center Hamburg-Eppendorf, Martinistr. 52, 20246 Hamburg, Germany; h.koenig@uke.de (H.-H.K.); j.dams@uke.de (J.D.)

**Keywords:** surveys and questionnaires, health care, utilization, migrant, Germany

## Abstract

There is ambiguous evidence with regard to the inequalities in health care services utilization (HCSU) among migrants and non-migrants in Germany. The aim of this study was to analyze the utilization of doctors and hospitalization of persons with direct and indirect migration background as well as those without in Germany. This study was based on data of the German Socio-Economic Panel using the adult sample of the years 2013 to 2019. HCSU was measured by self-reported utilization of doctors and hospitalization. Associations between HCSU and migration background were examined using multilevel mixed-effects logistic regression and zero-truncated multilevel mixed-effects generalized linear models. The odds ratios of utilization of doctors and hospitalization for persons with direct migration background compared with persons without migration background were 0.73 (*p* < 0.001) and 0.79 (*p* = 0.002), respectively. A direct migration background was associated with a 6% lower number of doctoral visits within three months compared with no migration background (*p* = 0.023). Persons with direct migration background still have a lower HCSU than persons without migration background in Germany. Access to health care needs to be ensured and health policy-makers are called upon to keep focus on the issue of inequalities in HCSU between migrants and non-migrants in Germany.

## 1. Introduction

In the year 2019, 26% of the total German population, about 21.2 million persons, had a migration background (based on a definition of migration background to people born without German nationality or if at least one parent was born without German nationality) [1]. Within the last ten years until 2019, this proportion of the population with migration background increased by 38%. Not only did the emergence of new migratory flows with the fall of the Iron Curtain and the expansion of the European Union in the last three decades lead to an increase in persons with direct migration background [2], but also the growing up of a new generation of persons, i.e., persons with indirect migration background, whose parents were work migrants, had bearing on this increase too [3,4].

A recent systematic literature review about inequalities in health care services utilization (HCSU) among migrants and non-migrants in Germany found a lower utilization among persons with migration background [5]. This lower utilization was shown, among others, for specialists, therapists, and medication, while the results for utilization of doctors and hospitalization were inconclusive. Furthermore, persons with direct migration background and females with migration background were identified as groups with a particular low HCSU. Another systematic literature review on HCSU of migrants in Europe; however, found that the probability of hospitalization of persons with migration background was higher compared with persons without migration background, whereas the results for utilization of doctors were also inconclusive [6].

According to the behavioral model by Andersen [7,8], HCSU is determined by predisposing, enabling, and need factors, where migration background might be seen as predisposing factor. Other predisposing factors are age, sex, and marital status; employment status is an enabling factor and health-related quality of life is a need factor that determines HCSU [7,8]. There is evidence that persons with direct migration background have a higher mental and lower physical health-related quality of life compared with persons without migration background in Germany [9,10,11], while no differences in health-related quality of life were observed for persons with indirect migration background [9,10,11,12,13]. Thus, differences in health-related quality of life might result in differences in HCSU of persons with and without migration background. It can, therefore, be assumed that persons with migration background have different needs and barriers with respect to health care than persons without migration background, and that migration background might not only be a predisposing factor, but also confounded with health and health-related quality of life, and other enabling and need factors [5,7,8]. Furthermore, it needs to be clarified whether the aforementioned assumptions are valid for both persons with direct migration background and for their descendants with indirect migration background [10,14,15].

In order to overcome the inconclusive results with respect to inequalities in HCSU and to follow the obstacles of differences in different needs and barriers with respect to health care among migrants and non-migrants in Germany, a study with a large nationally representative sample of persons with and without migration background is needed in order to be able to make a generally valid statement about the health care situation of migrants in Germany. However, earlier studies on the utilization of doctors and hospitalization of persons with and without migration background in Germany were based on regional samples [16,17,18,19,20,21,22,23,24,25,26,27,28], samples of children and adolescents or elderly [16,22,29,30,31,32,33,34,35], samples of women [18,19,20,21,24,25,26,27], or only samples on specific medical conditions [5,29,34,36,37]. Merely a study from 2011 by Glaesmer et al. [12] used a representative population survey of persons with direct and indirect migration background as well as those without to investigate differences in HCSU in Germany. The study, however, was not able to find any differences in the probability of utilization of doctors and hospitalization. Yet, a higher number of doctoral visits and nights in hospital of persons with direct migration background compared with persons without migration background was found.

The results of the aforementioned study should have called for a stronger health policy focus on access to health care services for persons with migration background. Because of those potentially unresolved issues, it is necessary to refocus research on HCSU of persons with migration background who have immigrated to Germany in the last three decades, but also on persons with indirect migration background. Based on the results of one earlier study [12], it is hypothesized that the migration background of more recently migrated persons and of those persons with indirect migration background is actually negatively associated with the probability of HCSU, and, if health care services were utilized, positively associated with the number of doctoral visits and nights in hospital. Therefore, the aim of this study was to analyze and compare the HCSU of persons with and without migration background in Germany. Thereby, the focus was on the utilization of doctors and hospitalization, as well as the number of doctoral visits and number of nights in hospital of persons with direct or indirect migration background and those without in a large representative sample.

## 2. Materials and Methods

### 2.1. Sample

The sample of this study was based on data of the German Socio-Economic Panel (SOEP) provided by the German Institute for Economic Research (DIW Berlin). The SOEP is a representative German household panel with over 20,000 participants surveyed annually since 1984, with 36 waves available up to 2021. As of the survey year 2013 (wave 29), two additional migrant samples (M1 and M2) were integrated into the SOEP to ensure the proportional representation of the previously underrepresented current generation of persons with migration background [38]. For the following analyses, the adult sample of the waves 29 to 36 (i.e., years 2013 to 2019) was used (*n* = 58,879; 251,930 observations). An analytical sample was generated by removing observations with missing information in the number of doctoral visits and number of nights in hospital (*n* = 44,403; 180,656 observations). Moreover, persons with missing information in sociodemographic characteristics were removed, resulting in a net sample of *n* = 43,921; 179,357 observations (75% of the original sample).

### 2.2. Measures

Persons without migration background and persons with direct/indirect migration background were distinguished based on a predefined variable of the SOEP. By combination of information on country of birth, citizenship, and of parental information, it was derived whether a person had an own migration experience or was born to at least one parent with direct migration background [39]. Concurrent with the definition of the European Migration Network [3,4], the DIW Berlin defined persons with direct migration background as persons with their own migration experience born without German citizenship, and persons with indirect migration background as persons without their own migration experience who were born to at least one parent with direct migration background [39]. Persons without migration background were persons born to parents without migration background.

In order to measure HCSU, participants of the SOEP were asked whether they had visited a doctor within the last three months and whether they had spent at least one night in hospital in the previous year. Furthermore, if they had visited a doctor within the last three months and if they had spent at least one night in hospital, they were asked how often they had visited a doctor within the last three months and how many nights in total they had spent in hospital within the last year, respectively. Regarding the utilization of doctors, no distinction was made in the SOEP between primary care physicians and specialists.

The sociodemographic characteristics age, sex (female and male), marital status (never married/single, married/in partnership, separated/divorced, and widowed), and employment status (employed fulltime, employed part-time, apprenticeship, marginally employed, other employment, and unemployed) were derived from the SOEP. For the purpose of illustration of the persons with direct/indirect migration background, nationality was also derived from the SOEP. Thereby, nationality was categorized into German, East European, South European, West and North European, African, Asian, and American/Oceanian countries of origin in accordance with the United Nations Standard Country or Area Codes for Statistical Use (M49) [40].

### 2.3. Statistical Analysis

Utilization of doctors within three months (yes/no) and hospitalization within the last year (yes/no) was dichotomized based on the questions on the utilization of a doctor within the last three months and on having spent at least one night in hospital within the last year. Furthermore, if persons utilized a doctor within the last three months, the number of doctoral visits within three months was used as a variable of HCSU. If persons spent at least one night in hospital within the last year, the number of nights in hospital within the last year was used.

Descriptive statistics of sociodemographic variables were calculated for persons without migration background and persons with direct and indirect migration background. Furthermore, differences in HCSU between persons without migration background and persons with direct or indirect migration background were calculated by sociodemographic characteristics (i.e., age, sex, marital status, employment status). The differences in HCSU by migration background were analyzed using Pearson’s chi-squared test and Student’s *t*-test. The descriptive statistics of sociodemographic variables and differences in HCSU were analyzed on the basis of cross-sectional data, using persons’ data at first occurrence in the selected analytical sample.

Associations between utilization of doctors within three months and hospitalization within one year and migration background were examined using multilevel mixed-effects logistic regression with cluster robust standard errors [41]. The group structure for the random effects was identified by a central individual identifier, which was fixed over time. Furthermore, the sociodemographic factors comprising age, sex, marital status, employment status, and survey year (2013 to 2019) were used. Furthermore, interactions between migration background and sex, and migration background and survey year were added to the models as independent variables. The fixed-effects coefficients of the logistic regressions were reported as odds ratios (OR).

The associations between the number of doctoral visits within three months, the number of nights in hospital within the last year, and migration background were examined using zero-truncated multilevel mixed-effects generalized linear models (GLM) with a negative binomial family and log link function [41]. For the GLM, the same group structure for the random effects, independent variables, and interactions as for the logistic regressions was taken into account. GLM with a negative binomial family take into account the skewed distribution and overdispersion of HCSU data as dependent variables [42]. The results of the GLM were reported as exponentiated fixed-effects coefficients.

All analyses were performed using Stata/SE 16.1 (StataCorp, College Station, TX, USA). All applied statistics were two-sided. The level of significance was set at α = 0.05.

## 3. Results

### 3.1. Sample Characteristics

The mean age of persons without migration background (*n* = 32,535) was 47 years. In comparison, persons with direct (*n* = 8080) and indirect migration background (*n* = 3306) were younger (42 and 30 years, both with *p* < 0.001). Of all persons without migration background and with indirect migration background, about half (52%) were female. Proportionally more persons with direct migration background were female (54%, *p* = 0.004). Persons with direct and indirect migration background differed in marital status and employment status (both with *p* < 0.001) compared with persons without migration background. Furthermore, the majority of persons with direct migration background had a German nationality (44%), followed by 18% and 16% with a nationality from a Southern European country and an Eastern European country, respectively. The vast majority of persons with indirect migration background had a German nationality (78%). The sociodemographic characteristics of the sample are shown in Table 1.

Of all persons without migration background, 72% (*n* = 23,510) utilized doctors within three months. Those who utilized doctors within three months had a mean number of doctoral visits of 2.38. Of all persons with direct and indirect migration background, 66% (*n* = 5342) and 67% (*n* = 2200) utilized doctors within three months, respectively. Those who utilized doctors within three months had mean numbers of doctoral visits of 2.05 and 2.07, respectively (Table S1 in the online Supplementary Materials).

Of all persons without migration background, 13% (*n* = 4282) were hospitalized within one year. Those who were hospitalized had a mean of 10.68 nights in hospital within one year. Of all persons with direct and indirect migration background, 10% were hospitalized within one year. Those who were hospitalized had a mean of 8.78 and 9.04 nights in hospital within one year, respectively (Table S2 in the online Supplementary Materials).

### 3.2. Doctoral Visits within Three Months

**Figure 1:**
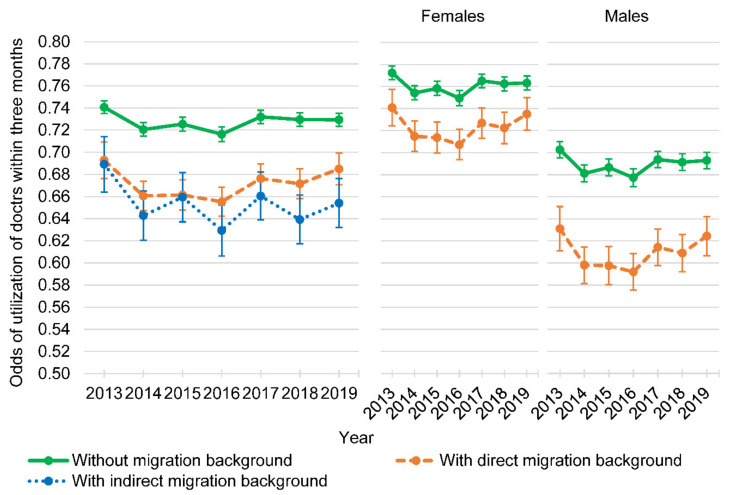
Adjusted odds of utilization of doctors within three months of persons without and with direct or indirect migration background, and for females and males without and with direct migration background (years 2013 to 2019, *n* = 43,921; 179,357 observations).

The logistic regression models showed that persons with direct migration background had lower odds of utilization of doctors within three months compared with persons without migration background (OR: 0.73, *p* < 0.001; Table 2). The odds ratio of the utilization of doctors within three months between persons with indirect migration background and persons without migration background was not statistically significant. Persons with a higher age (OR: 1.03; *p* < 0.001), females (OR: 1.60; *p* < 0.001), and persons not being employed fulltime (OR: 1.14–1.99; all with *p* < 0.001) had greater odds of hospitalization within one year in both models. Furthermore, the interaction of migration background and sex was statistically significant (*p* < 0.001) in the model comparing persons with direct and without migration background, indicating a modification of the association of direct migration background and the utilization of doctors by sex (Figure 1).

**Figure 2:**
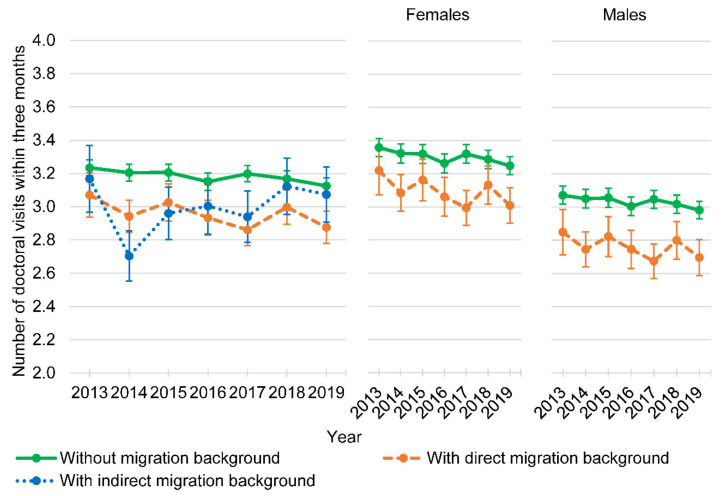
Adjusted zero-truncated number of doctoral visits within three months of persons without and with direct/indirect migration background, and of females and males without and with direct migration background (years 2013 to 2019, *n* = 31,052; 127,799 observations).

In the GLM, direct migration background was associated with a 6% reduction in the number of doctoral visits within three months compared with persons without migration background (*p* = 0.023), whereas no statistically significant association was found between indirect migration background and the number of doctoral visits within three months. The number of doctoral visits within three months was positively associated with a higher age (*p* < 0.001), female sex (*p* < 0.001), and not being employed fulltime or part-time (all with *p* ≤ 0.001) in the model comparing persons with direct and without migration background (all with *p* ≤ 0.001) and in the model comparing persons with indirect and without migration background (all with *p* ≤ 0.01). In the model comparing persons with direct and without migration background, the interaction of migration background and sex was statistically not significant (Figure 2).

No time trend was observed between the years 2013 and 2019 with regard to the utilization of doctors, nor with regard to the number of doctoral visits for any of the groups of persons analyzed.

### 3.3. Nights in Hospital within the Last Year

Persons with direct migration background had lower odds of hospitalization within one year than persons without migration background (OR: 0.79, *p* = 0.002; Table 3) in the logistic regression models. The odds ratio of hospitalization within one year of persons with indirect migration background and persons without migration background was not statistically significant. Persons with a higher age (OR: 1.01; *p* < 0.001) and persons not being employed fulltime or part-time (OR: 1.33–2.73; all with *p* < 0.001) had greater odds of hospitalization within one year in both models. Greater odds of hospitalization within one year associated with female sex (OR: 1.05; *p* < 0.001) were only found in the model analyzing differences between persons with direct and without migration background. Compared with being married or in a partnership, having never been married or being single were both associated with lower odds of hospitalization within one year in both models (OR 0.79 and 0.77; both with *p* < 0.001), whereas being separated, divorced, or widowed were associated with higher odds of hospitalization within one year in both models (OR 1.16–1.23; all with *p* < 0.001). Furthermore, in the model comparing persons with direct and without migration background, the interaction of migration background and sex was statistically significant (*p* < 0.001), indicating a modification of the association of direct migration background and hospitalization by sex (Figure S1 in the online Supplementary Materials).

In the GLM, no statistically significant associations were found between direct or indirect migration background and the number of nights in hospital within the last year. The number of nights in hospital within the last year was positively associated with a higher age (*p* < 0.001) and negatively associated with female sex (*p* = 0.012 and 0.017) in both models (Figure S2 in the online Supplementary Materials).

No time trend was observed between the years 2013 and 2019 with regard to hospitalization, or with regard to the number of nights in hospital for any of the groups of persons analyzed.

## 4. Discussion

### 4.1. Main Findings

The aim of this study was to analyze the HCSU of persons with direct and indirect migration background compared with persons without migration background in Germany. Persons with direct migration background had lower odds of utilization of doctors within three months than persons without migration background. Lower odds of utilization of doctors within three months were particularly observed in men with direct migration background. Furthermore, for persons utilizing doctors within three months, the number of doctoral visits was lower for persons with direct migration background compared with persons without migration background. For persons with indirect migration background, no differences in the odds of utilization of doctors and in the number of doctoral visits were found. Hence, only a direct migration background can be seen as predisposing factor for determining a lower utilization of doctors as well as a lower number of doctoral visits [7,8]. Consequently, direct migration background might still be associated with fewer need factors determining utilization of doctors, as persons with direct migration background were, on average, healthier than the German population without migration background. However, this effect might not occur in persons with indirect migration background in connection with the utilization of doctors. Furthermore, male sex of persons with direct background can be seen as a predisposing factor for determining a lower utilization of doctors. Yet, it cannot be ruled out that other unobserved determinants of HCSU, such as health and health-related quality of life, and other enabling and need factors have had an influence on direct migration background as predisposing factor for determining a lower utilization of doctors.

With regard to hospitalization, persons with direct migration background had lower odds of hospitalization within one year than persons without migration background. However, no difference was found among hospitalized persons with direct migration background in the number of nights in hospital. For persons with indirect migration background, no differences were found in the odds of hospitalization, nor in the number of nights in hospital compared with persons without migration background. Hence, a direct migration background can also be seen as predisposing factor for determining a lower hospitalization, yet not for the number of nights in hospital [7,8]. Likewise, for determining hospitalization, direct but not indirect migration background might also still be associated with fewer need factors, as persons with direct migration background were, on average, healthier than the German population without migration background. For other predisposing and enabling factors determining HCSU, such as age, marital status, and employment status, it was controlled for in the logistic regression models and in the GLM. However, no inferences can be drawn on those potential determinants of HCSU with regard to migration background based on the current analyses, as they were not added as interactions to the logistic regression models and GLM as independent variables due to a lack of statistical significance. Furthermore, there are other predisposing, enabling, and need factors that determine HCSU, such as education, socio-economic status, or health status, which were not controlled for in the models [7,8]. However, it is known that the perceived need of health care is explainable by education and health beliefs [8], and health status is associated with HCSU. Not considering those determinants as independent variables in the models might have led to omitted variable bias.

### 4.2. Previous Research and Possible Explanations

One earlier study that also used a representative population survey of persons with direct or indirect migration background as well as those without in Germany could not confirm migration background as a predisposing factor for determining the utilization of doctors and hospitalization [12]. The odds of the utilization of general practitioners were not statistically significantly different, but the odds of the utilization of specialists were statistically significantly lower for persons with direct migration background compared with persons without migration background (OR 0.58), even lower than the odds of utilization of doctors found in the current study (OR 0.73). With regard to the number of doctoral visits and the number of nights in hospital, the study by Glasemer et al. [12] found a lower number for persons with direct migration background compared with persons without migration background.

A systematic literature review on the HCSU of persons with and without migration background in Germany found evidence for an overall lower utilization of specialists for persons with direct migration background [5]. With respect to hospitalization, the evidence found in the review was inconclusive. This is somewhat consistent with the results of the current study, as only differences in the odds of hospitalization were found for persons with direct migration background compared with persons without migration background, but not for persons with indirect migration background, nor in the number of nights in hospital within the last year. Together with the results of the study by Glaesmer et al. [12] and the systematic literature review [5], the current study can confirm continuing disparities in HSCU, especially for persons with direct migration background and connected with the utilization of doctors.

The mechanisms of a lower HCSU, in particular of persons with direct migration background, are not conclusively resolved. One possible reason for differences in HCSU might be inequalities in cultural preferences and health beliefs [5]. Other reasons can be inequalities in access to health care, for example due to a lack of information or communication barriers, or a lack of management of cultural diversity by health care workers [5,43,44]. Accordingly, inequalities are to be reduced by the health policy makers and health care workers making sure that cultural stereotypes are minimized, that health communication is target-specific, and that language barriers are removed [5,45].

In contrast to this, sex is widely known to be a predisposing factor determining HCSU [7,8]; also, in the German health care system, women utilize doctors more often than men [46,47]. Previous research has shown that sex is also associated with HCSU for persons with migration background [48]. However, in the current study, the negative association of utilization of doctors and male sex was even stronger among persons with direct migration background compared with persons without migration background. Possible explanations for this disproportionally low utilization of doctors by men with direct migration background may be a major hurdle with regard to health care services or merely the alleged absence of occasions of visiting a doctor, such as the unawareness of the availability of free preventive check-ups. Another explanation might be the greater proximity of women to the health care system, e.g., through regular gynecological preventive and pregnancy check-ups, as well as the occurrence of maternity health problems. Furthermore, in the current study, women with direct migration background had higher odds of hospitalization within one year than women without migration background. One possible explanation for this might be a higher birth rate among women with direct migration background [49,50,51]. The open questions and assumptions made with regard to the disproportionally high utilization of doctors and hospitalization by females with migration background still need to be confirmed on the basis of data other than those from the SOEP, which include reasons of utilization.

### 4.3. Generalizability

The proportion of persons with migration background in the sample of this study was 26%. This proportion corresponds to the proportion of the total German population with migration background. It has to be noted that the integration of the two additional migrant samples into the SOEP ensured this proportional representation of persons with migration background [38]. As the data of the SOEP used in the current study were representative of German households, it can be assumed that the results of this study can be generalized to a certain extent to all adult persons with and without migration background in Germany. However, it has to be acknowledged that 25% of the adult sample that was used for the analysis was removed due to missing information in the number of doctoral visits and number of nights in hospital. Thereby, a disproportionately large number of persons with direct migration background was excluded from the analyses. Furthermore, the persons of the sample that were removed from the analysis due to missing information were younger and more likely to be male. Thus, generalizability may be limited.

Furthermore, it is possible that migrants with better German language skills and with better integration and education were more likely to be included in the SOEP. However, the questionnaires of the SOEP are available in multiple languages and were further translated if necessary [38]. Nevertheless, generalizability of results may, therefore, be further limited.

### 4.4. Strengths and Limitations

To our knowledge, this was the first analysis of HCSU of persons with direct or indirect migration background as well as those without in Germany, based on a large longitudinal sample. The major strength of this study was the use of data from a representative German household panel that recently integrated additional migrant samples to ensure a proportional representation of persons with migration background. The use of multilevel mixed-effects logistic regressions and GLM with a negative binomial family can also be considered as strength of this study.

However, this study has some limitations that should be mentioned. First, HCSU might be biased due to seasonal effects of utilization of doctors, as the number of doctoral visits were inquired retrospectively only within a period of three months. Second, no migration-specific characteristics, i.e., years since arrival in Germany, language skills, or connection to Germany, were used as explanatory variables in the analyses. Since these variables are most likely to be correlated with migration background and since multicollinearity should be avoided in regression models, it can be assumed that these variables would have been excluded from the models anyway. Third, mixed-effects GLM with truncated zero values were used to analyze the associations between the number of doctoral visits within three months, the number of nights in hospital within the last year, and migration background. However, for zero-truncated data, a zero-truncated negative binomial model would have been more appropriate. Unfortunately, such model is not yet implemented for mixed-effects in Stata [42].

## 5. Conclusions

Persons with direct migration background still have lower odds of utilization of health care services than persons without migration background in Germany. Here, not only were odds of doctor utilization and hospitalization lower, but also the number of doctoral visits. Fortunately, no differences in the utilization of health care services were found for persons with indirect migration background. The call for a stronger health policy focus on access to health care for persons with direct migration background, especially for men, remains relevant with regard to the results of the present study. In addition, further research is needed to better understand the underlying causes and reasons for reduced HCSU of persons with direct migration background.

## Figures and Tables

**Table 1:** Sociodemographic characteristics (years 2013 to 2019, *n* = 43,921).

Sociodemographic Characteristic	Persons without Migration Background (*n* = 32,535)	Persons with Direct Migration Background (*n* = 8080)	Persons with Indirect Migration Background (*n* = 3306)
Age: Mean (SD)	47.16 (17.98)	42.00 (14.16) ***	30.17 (12.07) ***
Grouped age: *n* (%)			
18–24	4641 (14.26)	773 (9.57) ***	1465 (44.31) ***
25–34	4038 (12.41)	1864 (23.07)	729 (22.05)
35–44	5573 (17.13)	2364 (29.26)	678 (20.51)
45–54	7123 (21.89)	1555 (19.25)	289 (8.74)
55–64	5002 (15.37)	905 (11.20)	113 (3.42)
≥65	6158 (18.93)	619 (7.66)	32 (0.97)
Sex: *n* (%)			
Female	16,892 (51.92)	4338 (53.69) **	1713 (51.81)
Male	15,643 (48.08)	3742 (46.31)	1593 (48.19)
Marital status: *n* (%)			
Never married/single	9754 (29.98)	1661 (20.56) ***	1990 (60.19) ***
Married/in partnership	17,364 (53.37)	5386 (66.66)	1085 (32.82)
Separated/divorced	3779 (11.62)	838 (10.37)	211 (6.38)
Widowed	1638 (5.03)	195 (2.41)	20 (0.60)
Employment status: *n* (%)			
Employed fulltime	12,691 (39.01)	3171 (39.25) ***	1106 (33.45) ***
Employed part-time	4635 (14.25)	1075 (13.30)	346 (10.47)
Apprenticeship	1110 (3.41)	191 (2.36)	297 (8.98)
Marginally employed	1952 (6.00)	656 (8.12)	289 (8.74)
Other employment ^1^	290 (0.89)	21 (0.26)	33 (1.00)
Unemployed	11,857 (36.44)	2966 (36.71)	1235 (37.36)
Nationality: *n* (%)			
German	32,535 (100.00)	3573 (44.22) ***	2573 (77.83) ***
East European	-	1419 (17.56)	10 (0.30)
South European	-	1260 (15.59)	376 (11.37)
West and North European ^2^	-	382 (4.73)	43 (1.30)
African	-	178 (2.20)	9 (0.27)
Asian	-	1123 (13.90)	286 (8.65)
American/Oceanian	-	128 (1.58)	8 (0.24)
Stateless/ethnic minority	-	17 (0.21)	1 (0.03)

Comments: SD: Standard deviation; ** *p* ≤ 0.01, *** *p* ≤ 0.001; comparison of categorical characteristics of persons without migration background and with direct/indirect migration background was analyzed using Pearson’s chi-squared test; comparison of mean age of persons without migration background and with direct/indirect migration background was analyzed using Student’s *t*-test; ^1^ Near retirement with zero working hours, military service, community service, sheltered workshop; ^2^ Without German nationality.

**Table 2:** Multilevel mixed-effects logistic regressions of doctoral visits within three months (yes/no) and zero-truncated multilevel mixed-effects generalized linear models of number of doctoral visits within three months for persons without and with direct or indirect migration background (years 2013 to 2019, *n* = 43,921; 179,357 observations).

Independent Variables	Without Migration Background vs. with Direct Migration Background		Without Migration Background vs. with Indirect Migration Background	
	OR (SE) ^†^	Exp(β) (SE) ^‡^	OR (SE) ^†^	Exp(β) (SE) ^‡^
Migration background (Ref. without migration background)				
With direct migration background	0.73 (0.05) ***	0.94 (0.02) *		
With indirect migration background			0.99 (0.09)	0.99 (0.04)
Age	1.03 (0.00) ***	1.00 (0.00) ***	1.03 (0.00) ***	1.00 (0.00) ***
Sex (Ref. male)				
Female	1.60 (0.04) ***	1.08 (0.01) ***	1.60 (0.04) ***	1.08 (0.01) ***
Marital status (Ref. married/in partnership)				
Never married/single	1.16 (0.03) ***	0.98 (0.01)	1.18 (0.04) ***	0.97 (0.01) *
Separated/divorced	1.03 (0.03)	1.08 (0.01) ***	1.00 (0.04)	1.08 (0.01) ***
Widowed	1.02 (0.06)	1.00 (0.02)	1.04 (0.06)	0.99 (0.02)
Employment status (Ref. employed fulltime)				
Employed part-time	1.14 (0.03) ***	1.00 (0.01)	1.15 (0.03) ***	1.00 (0.01)
Apprenticeship	1.99 (0.11) ***	1.07 (0.02) ***	1.94 (0.10) ***	1.05 (0.02) **
Marginally employed	1.25 (0.04) ***	1.06 (0.01) ***	1.26 (0.05) ***	1.06 (0.01) ***
Other employment ^1^	1.88 (0.20) ***	1.12 (0.04) ***	1.78 (0.19) ***	1.10 (0.04) **
Unemployed	1.70 (0.04) ***	1.24 (0.01) ***	1.65 (0.04) ***	1.22 (0.01) ***
Migration background * Sex (Ref. no migration background * male)				
Direct migration background * Female	1.21 (0.06) ***	1.01 (0.02)		
Indirect migration background * Female			1.32 (0.10) ***	1.08 (0.03) **
Survey year	Yes	Yes	Yes	Yes
Migration background * Survey year	Yes	Yes	Yes	Yes
Constant	0.48 (0.02) ***	2.24 (0.04) ***	0.49 (0.03) ***	2.27 (0.04) ***
Random effect: Person-ID				
Variance (Constant)	2.12 (0.04)	0.24 (0.00)	2.16 (0.04)	0.24 (0.00)

Comments: CI: confidence interval; SE: standard error; ^1^ near retirement with zero working hours, military service, community service, sheltered workshop; ^†^ Dependent variable: utilization of doctors within three months (yes/no); ^‡^ Dependent variable: number of doctoral visits (*n* = 31,052; 127,799 observations); * *p* ≤ 0.05, ** *p* ≤ 0.01, *** *p* ≤ 0.001.

**Table 3:** Multilevel mixed-effects logistic regressions of hospitalization (yes/no) and zero-truncated multilevel mixed-effects generalized linear models of number of nights in hospital within the last year for persons without and with direct or indirect migration background (years 2013 to 2019, *n* = 43,921; 179,357 observations).

Independent Variables	Without Migration Background vs. with Direct Migration Background		Without Migration Background vs. with Indirect Migration Background	
	OR (SE) ^†^	Exp(β) (SE) ^‡^	OR (SE) ^†^	Exp(β) (SE) ^‡^
Migration background (Ref. without migration background)				
With direct migration background	0.79 (0.06) **	0.93 (0.06)		
With indirect migration background			0.82 (0.10)	1.16 (0.15)
Age	1.01 (0.00) ***	1.01 (0.00) ***	1.01 (0.00) ***	1.01 (0.00) ***
Sex (Ref. male)				
Female	1.05 (0.03) *	0.95 (0.02) *	1.05 (0.03)	0.95 (0.02) *
Marital status (Ref. married/in partnership)				
Never married/single	0.79 (0.03) ***	1.06 (0.03) *	0.77 (0.03) ***	1.05 (0.03)
Separated/divorced	1.23 (0.04) ***	1.17 (0.03) *	1.19 (0.04) ***	1.19 (0.03)
Widowed	1.17 (0.05) ***	1.09 (0.03) ***	1.16 (0.05) ***	1.06 (0.03) ***
Employment status (Ref. employed fulltime)				
Employed part-time	1.03 (0.04)	1.02 (0.03)	1.06 (0.04)	1.02 (0.03)
Apprenticeship	1.54 (0.11) ***	1.15 (0.09)	1.64 (0.11) ***	1.24 (0.09) **
Marginally employed	1.33 (0.06) ***	1.08 (0.04)	1.38 (0.07) ***	1.06 (0.05)
Other employment ^1^	1.90 (0.22) ***	1.15 (0.12)	1.83 (0.22) ***	1.09 (0.11)
Unemployed	2.73 (0.07) ***	1.34 (0.03) ***	2.68 (0.08) ***	1.33 (0.03) ***
Migration background * Sex (Ref. no migration background * male)				
Direct migration background * Female	1.21 (0.07) ***	1.00 (0.05)	1.40 (0.13) ***	1.03 (0.09)
Survey year	Yes	Yes	Yes	Yes
Migration background * Survey year	Yes	Yes	Yes	Yes
Constant	0.04 (0.00) ***	3.53 (0.15) ***	0.04 (0.00) ***	3.58 (0.16) ***
Random effect: Person-ID				
Variance(Constant)	1.32 (0.04)	0.46 (0.01)	1.35 (0.04)	0.47 (0.01)

Comments: CI: confidence interval; OR: odds ratio; SE: standard error; ^1^ near retirement with zero working hours, military service, community service, sheltered workshop; ^†^ dependent variable: hospitalization within one year (yes/no); ^‡^ dependent variable: number of nights in hospital (*n* = 5464; 23,421 observations); * *p* ≤ 0.05, ** *p* ≤ 0.01, *** *p* ≤ 0.001.

## Data Availability

The code used during the current study is available from the corresponding author on reasonable request for all interested researchers. Interested parties may contact the Department of Health Economics and Health Services Research, University Medical Center Hamburg-Eppendorf (contact information: Thomas Grochtdreis, t.grochtdreis@uke.de, +49-40-7410–52405).

## Supplementary Materials

The following are available online at https://www.mdpi.com/article/10.3390/ijerph182111640/s1; Table S1: Number of persons with utilization of doctors within three months and zero-truncated mean number of doctoral visits within three months by sociodemographic characteristics and migration background (waves 2013 to 2019, *n* = 43,921); Table S2: Number of persons with hospitalization and zero-truncated mean number of nights in hospital within the last year by sociodemographic characteristics and migration background (waves 2013 to 2019, *n* = 43,921); Figure S1: Adjusted odds of hospitalization within one year of persons without and with direct/indirect migration background, and of females and males without and with direct migration background (waves 2013 to 2019, *n* = 43,921; 179,357 observations); Figure S2: Adjusted zero-truncated number of nights in hospital within the last year of persons without and with direct/indirect migration background, and of females and males without and with direct migration background (waves 2013 to 2019, *n* = 5464; 23,421 observations).
