# Supplementary material for: Health Care Services Utilization of Persons with Direct, Indirect and without Migration Background in Germany: A Longitudinal Study Based on the German Socio-Economic Panel (SOEP)
Source: Int J Environ Res Public Health. 2021 Nov 5;18(21):11640. doi: 10.3390/ijerph182111640 (PMC8583515; doi:10.3390/ijerph182111640)
Supplement: Supplementary file 1 [file ijerph-18-11640-s001.zip › Table S1_Doctoral visits.pdf]

Table S1. Number of persons with utilization of doctors and number of doctoral visits within three months

Sociodemographic characteristic	Persons without migration background (n=32535)		Persons with direct migration background (n=8080)		Persons with indirect migration background (n=3306)	
	n (%)	Mean (SE)	n (%)	Mean (SE)	n (%)	Mean (SE)
Total sample	23510 (72.26)	2.38 (0.02)	5342 (66.11)	2.05 (0.04)	2200 (66.55)	2.07 (0.06)
Grouped age						
18 – 24	3049 (65.70)***	1.84 (0.05)***	460 (59.51)***	1.63 (0.12)***	971 (66.28)	2.02 (0.08)
25 – 34	2634 (65.23)	2.08 (0.06)	1138 (61.05)	1.76 (0.08)	474 (65.02)	1.96 (0.12)
35 – 44	3723 (66.80)	2.13 (0.05)	1494 (63.20)	1.93 (0.07)	452 (66.67)	2.01 (0.12)
45 – 54	4979 (69.90)	2.34 (0.04)	1059 (68.10)	2.27 (0.09)	196 (67.82)	2.59 (0.19)
55 – 64	3849 (76.95)	2.61 (0.05)	683 (75.47)	2.52 (0.11)	82 (72.57)	2.27 (0.30)
≥65	5276 (85.68)	3.08 (0.05)	508 (82.07)	2.69 (0.14)	25 (78.13)	2.28 (0.56)
Sex						
Female	12825 (75.92)***	2.60 (0.03)***	3122 (71.97)***	1.70 (0.06)***	1247 (72.80)***	1.65 (0.08)***
Male	10685 (68.31)	2.14 (0.03)	2220 (59.33)	2.36 (0.05)	953 (59.82)	2.46 (0.08)
Marital status						
Never married/single	6510 (66.74)***	2.04 (0.04)***	989 (59.54)***	1.71 (0.08)***	1305 (65.58)*	1.92 (0.07)***
Married/in partnership	12811 (73.78)	2.41 (0.03)	3602 (66.88)	2.02 (0.05)	719 (66.27)	2.10 (0.10)
Separated/divorced	2806 (74.25)	2.81 (0.06)	597 (71.24)	2.65 (0.12)	160 (75.83)	3.27 (0.22)
Widowed	1383 (84.43)	3.05 (0.09)	154 (78.97)	3.38 (0.24)	16 (80.00)	2.50 (0.71)
Employment status						
Employed fulltime	8413 (66.29)***	1.92 (0.03)***	1887 (59.51)***	1.50 (0.06)***	689 (62.30)**	1.67 (0.10)***
Employed part-time	3358 (72.45)	2.24 (0.05)	697 (64.84)	1.77 (0.10)	233 (67.34)	2.37 (0.17)
Apprenticeship	756 (68.11)	1.88 (0.11)	126 (65.97)	2.07 (0.24)	203 (68.35)	1.94 (0.18)
Marginally employed	1410 (72.23)	2.30 (0.08)	436 (66.46)	1.88 (0.13)	211 (73.01)	2.014 (0.19)
Other employment[1]	228 (78.62)	2.52 (0.22)	17 (80.95)	1.67 (0.74)	24 (72.73)	1.73 (0.55)
Unemployed	9345 (78.81)	2.99 (0.03)	2179 (73.47)	2.79 (0.06)	840 (68.02)	2.39 (0.09)

Comments: Years 2013 to 2019, n=43,921; SE: Standard error; comparison of utilization of doctors within three months by categorical characteristics was analyzed using Pearson's chi-squared test; comparison of zero-truncated mean annual number of doctoral visits by categorical characteristics was analyzed using Student's t-test; [1] Near retirement with zero working hours, military service, community service, sheltered workshop; * $p \leq 0.05$, ** $p \leq 0.01$, *** $p \leq 0.001$.
