# Supplementary material for: Health Care Services Utilization of Persons with Direct, Indirect and without Migration Background in Germany: A Longitudinal Study Based on the German Socio-Economic Panel (SOEP)
Source: Int J Environ Res Public Health. 2021 Nov 5;18(21):11640. doi: 10.3390/ijerph182111640 (PMC8583515; doi:10.3390/ijerph182111640)
Supplement: Supplementary file 1 [file ijerph-18-11640-s001.zip › Table S2_Hospitalization.pdf]

Table S2. Number of persons with hospitalization within one year and number of annual nights in hospital

Sociodemographic characteristic	Persons without migration background (n=32535)		Persons with direct migration background (n=8080)		Persons with indirect migration background (n=3306)	
	**n (%)**	**Mean (SE)**	**n (%)**	**Mean (SE)**	**n (%)**	**Mean (SE)**
Total sample	4282 (13.16)	10.68 (0.13)	841 (10.41)	8.78 (0.29)	341 (10.31)	9.04 (0.49)
Grouped age						
18 – 24	469 (10.11)***	7.09 (0.52)***	65 (8.41)***	6.84 (1.15)***	145 (9.90)**	8.38 (1.10)***
25 – 34	502 (12.43)	7.16 (0.41)	205 (11.00)	6.27 (0.56)	63 (8.64)	6.65 (1.20)
35 – 44	588 (10.55)	8.34 (0.37)	219 (9.26)	7.31 (0.50)	71 (10.47)	7.72 (1.15)
45 – 54	722 (10.14)	10.65 (0.31)	142 (9.13)	8.81 (0.54)	36 (12.46)	13.32 (1.88)
55 – 64	695 (13.89)	11.40 (0.31)	97 (10.72)	10.04 (0.63)	17 (15.04)	20.90 (2.51)
≥65	1306 (21.21)	12.52 (0.21)	113 (18.26)	13.19 (0.61)	9 (28.13)	11.2 (4.77)
Sex						
Female	2312 (13.69)**	10.26 (0.17)***	524 (12.08)***	8.35 (0.32)*	211 (12.32)***	9.24 (0.76)***
Male	1970 (12.59)	11.20 (0.19)	317 (8.47)	9.45 (0.40)	130 (8.16)	8.66 (1.01)
Marital status						
Never married/single	1009 (10.34)***	9.30 (0.31)***	157 (9.45)***	7.13 (0.69)***	182 (9.15)***	8.49 (0.86)***
Married/in partnership	2383 (13.72)	10.31 (0.17)	533 (9.90)	8.30 (0.30)	125 (11.52)	7.07 (0.90)
Separated/divorced	553 (14.63)	12.27 (0.34)	115 (13.72)	9.73 (0.65)	27 (12.80)	14.70 (2.02)
Widowed	337 (20.57)	12.95 (0.40)	36 (18.46)	17.25 (1.14)	7 (35.00)	49.37 (4.79)
Employment status						
Employed fulltime	1160 (9.14)***	8.00 (0.26)***	217 (6.84)***	6.43 (0.49)***	77 (6.96)***	7.42 (1.18)
Employed part-time	477 (10.29)	7.39 (0.38)	75 (6.98)	6.66 (0.76)	37 (10.69)	8.91 (1.67)
Apprenticeship	108 (9.73)	8.56 (0.98)	14 (7.33)	8.31 (2.28)	30 (10.10)	10.62 (2.31)
Marginally employed	233 (11.94)	9.06 (0.59)	51 (7.77)	7.11 (0.98)	24 (8.30)	7.24 (2.16)
Other employment[1]	39 (13.45)	8.42 (1.54)	3 (14.29)	16.00 (4.67)	3 (9.09)	5.43 (8.15)
Unemployed	2265 (19.10)	12.69 (0.17)	481 (16.22)	10.41 (0.33)	170 (13.77)	10.13 (0.91)

Legend: Years 2013 to 2019, n=43,921; SE: Standard error; comparison of hospitalization within one year by categorical characteristics was analyzed using Pearson's chi-squared test; comparison of zero-truncated mean annual nights in hospital by categorical characteristics was analyzed using Student's t-test; [1] Near retirement with zero working hours, military service, community service, sheltered workshop; * $p \leq 0.05$, ** $p \leq 0.01$, *** $p \leq 0.001$.
