# Supplementary figures and images for: Health Care Services Utilization of Persons with Direct, Indirect and without Migration Background in Germany: A Longitudinal Study Based on the German Socio-Economic Panel (SOEP)
Source: Int J Environ Res Public Health. 2021 Nov 5;18(21):11640. doi: 10.3390/ijerph182111640 (PMC8583515; doi:10.3390/ijerph182111640)

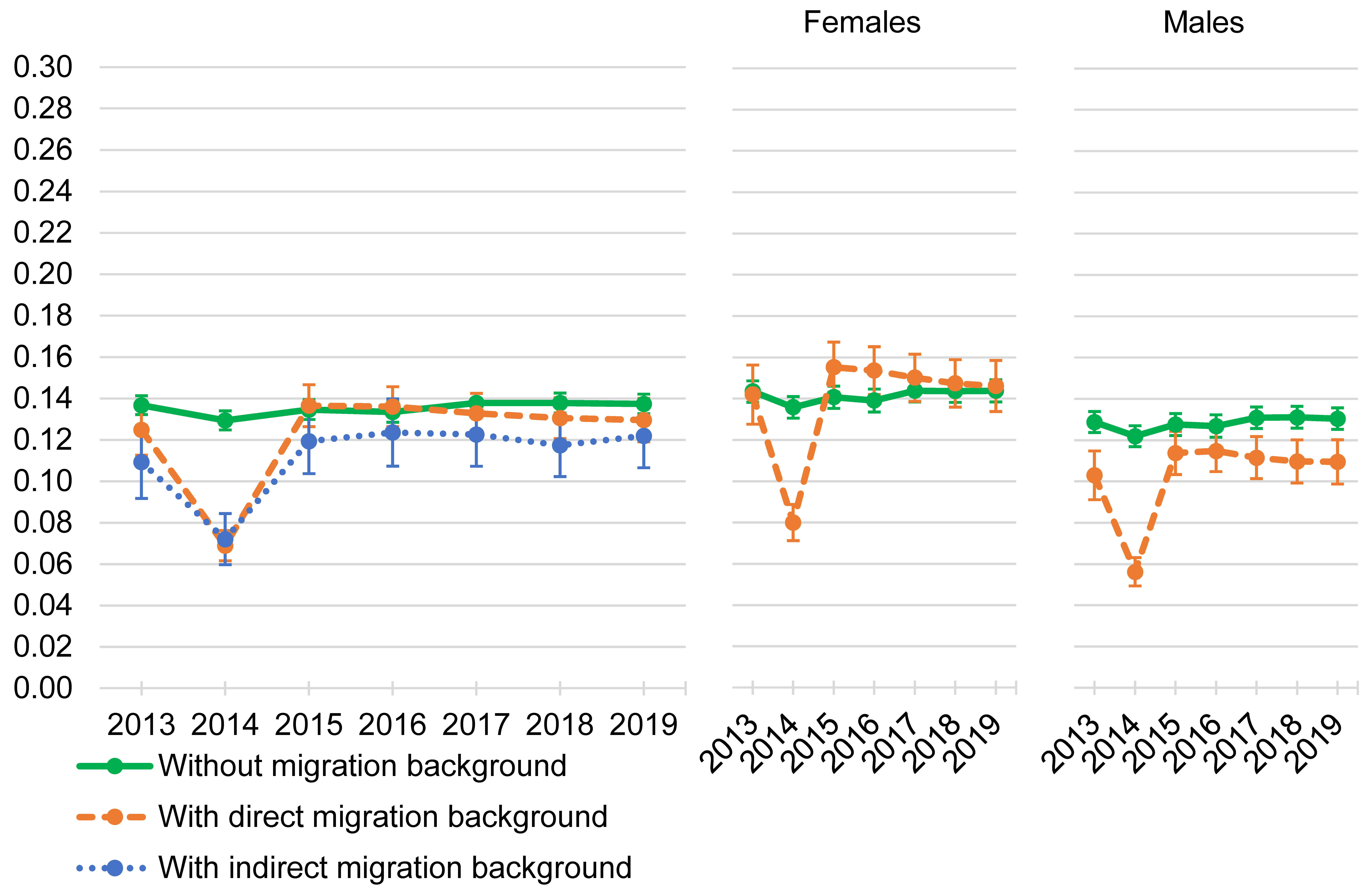

Supplement: Supplementary file 1 [file ijerph-18-11640-s001.zip › Figure_S1_Hospitalization_OR.tiff]

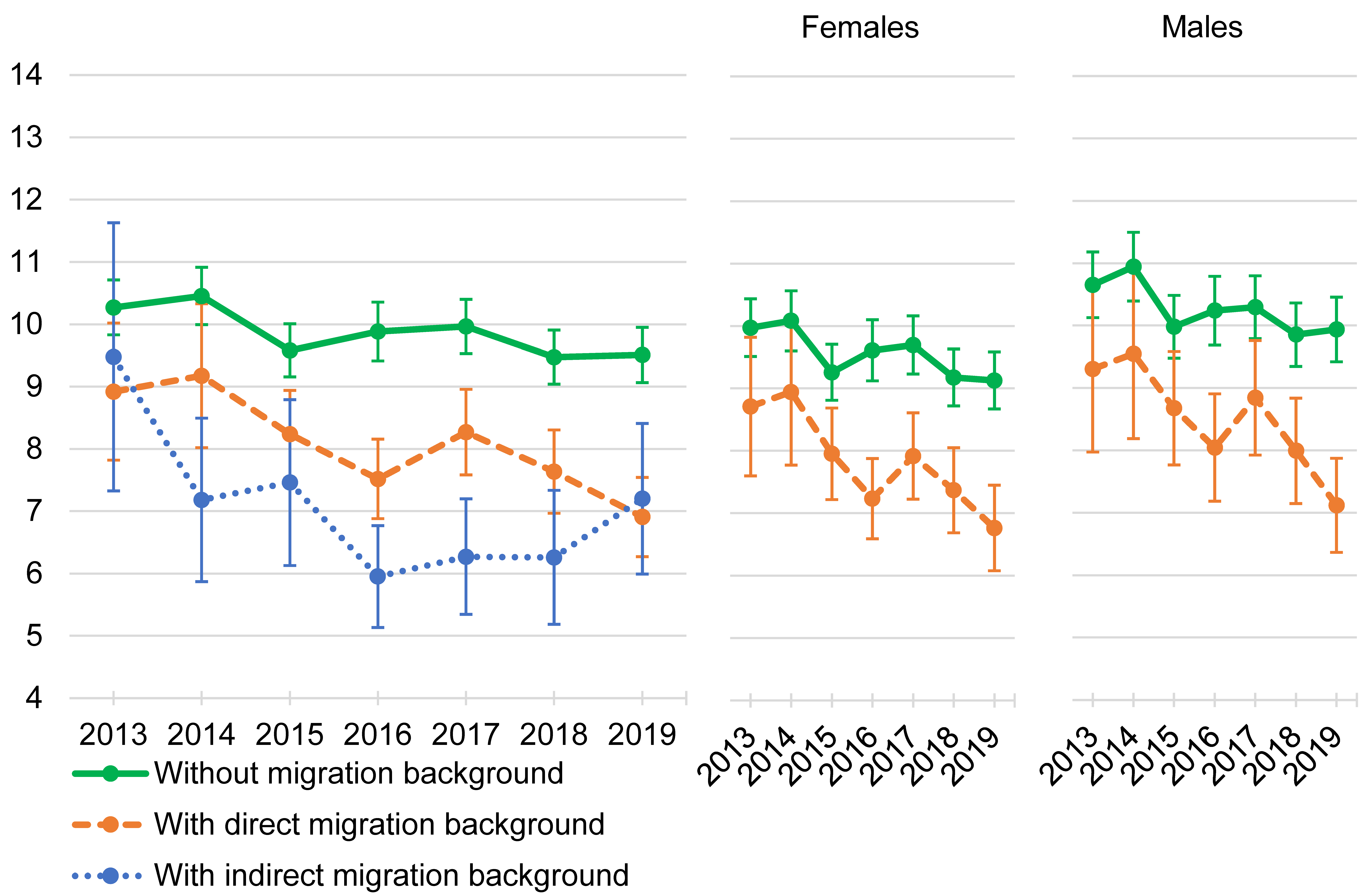

Supplement: Supplementary file 1 [file ijerph-18-11640-s001.zip › Figure_S2_Hospitalization_Reg.tiff]
